# Assessment of temperature and time on the survivability of porcine reproductive and respiratory syndrome virus (PRRSV) and porcine epidemic diarrhea virus (PEDV) on experimentally contaminated surfaces

**DOI:** 10.1371/journal.pone.0291181

**Published:** 2024-01-19

**Authors:** Mafalda Mil-Homens, Ethan Aljets, Rodrigo C. Paiva, Isadora Machado, Guilherme Cezar, Onyekachukwu Osemeke, Daniel Moraes, Swaminathan Jayaraman, Mckenna Brinning, Ana Paula Poeta Silva, Lauren Tidgren, Madison Durflinger, Mallory Wilhelm, Vivian Flores, Jolie Frenier, Daniel Linhares, Jianqiang Zhang, Derald Holtkamp, Gustavo S. Silva

**Affiliations:** Veterinary Diagnostic and Production Animal Medicine, College of Veterinary Medicine, Iowa State University, Ames, IA, United States of America; Plum Island Animal Disease Center, UNITED STATES

## Abstract

Fomites might be responsible for virus introduction in swine farms, highlighting the importance of implementing practices to minimize the probability of virus introduction. The study’s objective was to assess the efficacy of different combinations of temperatures and holding-times on detecting live PRRSV and PEDV on surfaces commonly found in supply entry rooms in swine farms. Two PRRSV isolates (MN 184 and 1-4-4 L1C variant) and one PEDV isolate (NC 49469/2013) were inoculated on cardboard and aluminum. An experimental study tested combinations of four temperatures (20°C, 30°C, 40°C, and 50°C) and six holding-times (15 minutes, 60 minutes, 6 hours, 12 hours, 24 hours, and 36 hours) for the presence of the viruses on each surface type. After virus titration, virus presence was assessed by assessing the cytopathic effects and immunofluorescence staining. The titers were expressed as log_10_ TCID_50_/ml, and regression models; half-lives equations were calculated to assess differences between treatments and time to not detect the live virus. The results suggest that the minimum time that surfaces should be held to not detect the virus at 30°C was 24 hours, 40°C required 12 hours, and 50°C required 6 hours; aluminum surfaces took longer to reach the desired temperature compared to cardboard. The results suggest that PRRSV 1-4-4 L1C variant had higher half-lives at higher temperatures than PRRSV MN 184. In conclusion, time and temperature combinations effectively decrease the concentration of PRRSV and PEDV on different surfaces found in supply entry rooms in swine farms.

## Introduction

Introducing fomites in swine farms via a supply entry room can be an indirect transmission route for various swine pathogens [1, 2]. A common practice in swine farms is the application of chemical disinfectants through fogging, although the use of foggers has the disadvantage of not reaching all surfaces and having poor results when the surface has organic matter [3]. An alternative method to disinfectants is holding surfaces at different combinations of time and temperature, as shown by [4–6]. Time and temperature have been evaluated in studies that simulate the heating of feed and trailers [5, 7–10], but there is a gap in knowledge about the application of combinations of time and temperature suitable for supply entry rooms under field conditions. Porcine reproductive and respiratory syndrome virus (PRRSV) and porcine epidemic diarrhea virus (PEDV) are of critical importance to control for modern swine production. PRRSV is associated with high mortality, decreased growth rate, systemic disease, and reproductive losses [1]. Its annual economic impact is estimated to be $664 million in the United States [11]. PRRSV was shown to survive for more than four months at low temperatures (−20 to −70°C), according to [12], however can be inactivated by washing, disinfecting, and drying for 12 hours at 20°C [8]. In addition, through thermo-assisted drying and decontamination method (TADD) in trailers, PRRSV was inactivated at 70°C for 120 minutes [9].

PEDV is associated with high neonatal mortality and morbidity in suckling piglets [13]. The annual economic impact was estimated to be $900 million to $1.8 billion in the United States [14]. PEDV can survive for one week at 20°C in dried plasma [15] and for more than seven days in feces [16]. Holding metal surfaces contaminated with PEDV at 71°C for 10 minutes using TADD was shown to be effective in PEDV inactivation [6].

It is crucial to assess combinations of temperature and time on contaminated fomites coming into the farm that can be applied in field conditions. Therefore, the objective of this study was to assess the efficacy of temperatures and holding-times on the survivability of PRRSV and PEDV on cardboard and diamond plate aluminum surfaces commonly found in the supply entry rooms in swine farms.

## Materials and methods

### Study design

**Fig 1:**
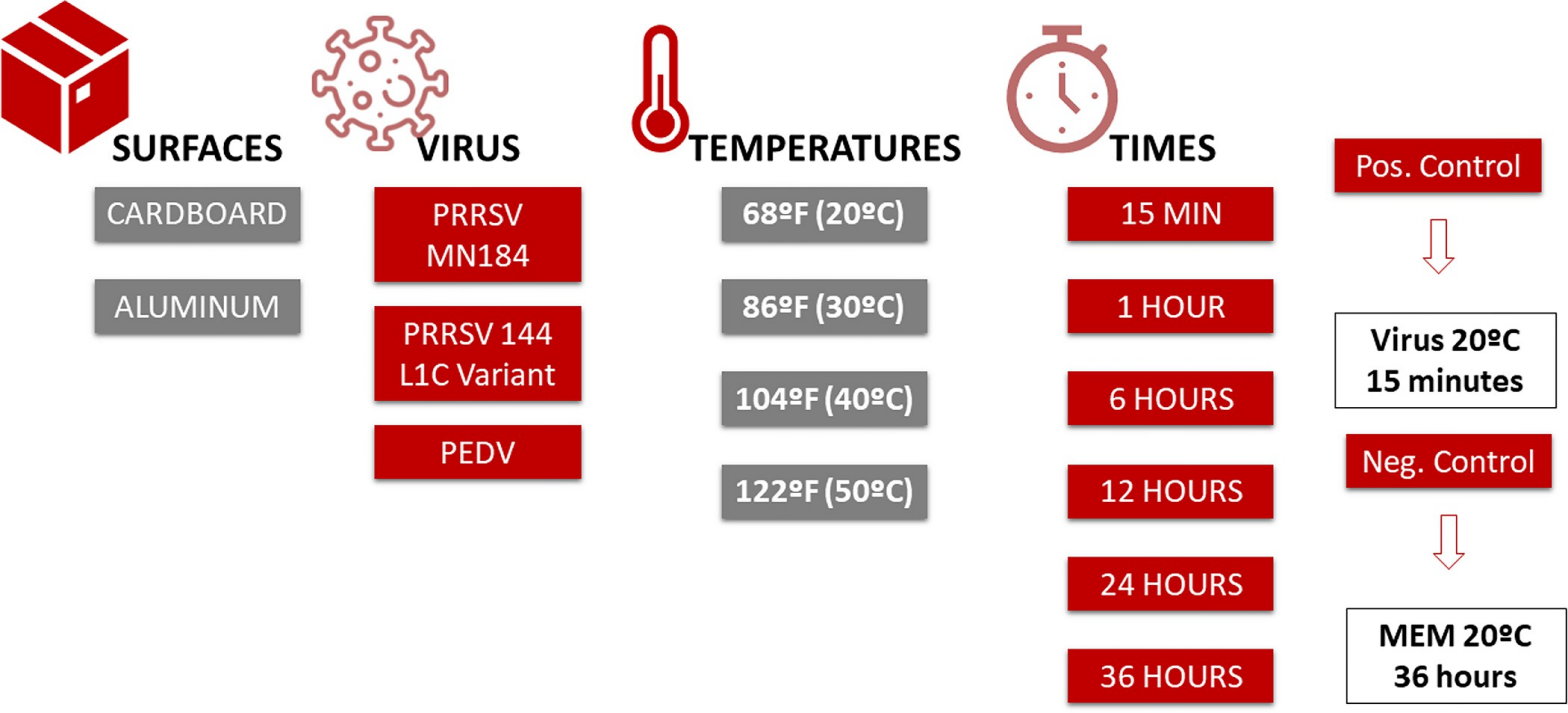
Study design. Description of the surfaces, viruses, temperatures and times evaluated in this study.

An experimental study was conducted to assess the effect of time and temperature on detecting PRRSV and PEDV isolates on two surface types: 1) diamond plate aluminum (Southside Welding and Machine LLC, Audubon, Iowa); 2) cardboard coupon measuring 7.62 cm x 7.62 cm x 1.27 cm. PRRSV and PEDV inactivation were tested at four temperature treatments (20°C, 30°C, 40°C, and 50°C) and six holding-time treatments (15 minutes, 60 minutes, 6 hours, 12 hours, 24 hours, and 36 hours). The negative control was held at 20°C for 36 hours. All treatments and negative control had three replicates. The treatments at 20°C for 15 minutes were used as positive controls. The study had a total of 144 treatments (2 surfaces x 3 viruses x 4 temperatures x 6 holding-times); 432 replicates contained live viruses while six replicates were without viruses (negative controls), totalizing 438 replicates (Fig 1). All procedures were conducted with the approval of the Iowa State University Office for Responsible Research (IBC Log #19–085) in a BSL-2 livestock infectious disease isolation facility equipped with a single-pass non-recirculating ventilation system for the prevention of aerosol cross-contamination.

### Virus isolates

The U.S. non-S INDEL (U.S. prototype) PEDV isolate USA/NC49469/2013 [17] was used in this study. PEDV has propagated in Vero (ATCC CCL-81) cells, as previously described by [18]. PRRSV MN 184 and 1-4-4 L1C variant ISU21-1775 isolates used in this study were propagated in MARC-145 (M-145) cells as described by [10].

### Coupon inoculation

Coupons were inoculated with 2 ml of PRRS virus (MN 184 and 1-4-4 L1C variant) with initial concentrations of 5.6 x 10⁵ log_10_TCID_50_/ml and 5.6 x 10⁴ log_10_TCID_50_/ml for PRRSV MN 184 and PRRSV 1-4-4 L1C variant, respectively. Coupons were inoculated with 2 ml of PEDV virus with an initial concentration of 3.16 x 10⁵ log_10_TCID_50_/ml. Negative controls were inoculated with 2 ml of Minimum Essential Medium (MEM; SIGMA, United States). In brief, coupons for each treatment were placed on aluminum turkey roasting trays, with three replicates per tray. The stock virus (PRRSV MN 184, PRRSV 1-4-4 LIC Variant or PEDV) or MEM for negative controls was pipetted onto the upper surface of each coupon taking care not to allow any liquid to run off the edges of the coupon, and the surface was scraped with a pipette to disperse the virus over the top surface of the coupons. Gloves and plastic covering the workbench were changed between each virus.

### Incubation period

Temperature and time combinations, as described above, were applied according to the treatment. Infrared thermometers (Eventek, United States of America) were used to monitor the surface temperature of the coupons for the 20°C treatment groups, and the negative control replicates that were kept at 20°C and approximately 30% relative humidity for the designated time.

A Fisherbrand Isotemp 400 incubator (Fisher Scientific, Chicago, Illinois) was used for the 30°C, 40°C, and 50°C treatment groups, and thermometers with K-Type thermocouple sensors (Perfect Prime TC41, United States of America) were used to monitor the surface temperature of the coupons placed inside the incubator. For the aluminum surface, the probe was taped on the edge of the surface. A small square on the inoculation area for the cardboard surface was left without virus. A small hole was made in that area of the cardboard with the tip of the thermocouple. The probe was inserted and taped to the cardboard surface. The contact time started once the surface reached the desired temperature.

### Sample collection

To recover the virus from the surfaces, 5 ml of MEM was placed inside a Petri dish, then 1 ml of MEM was eluted over the surface with a disposable pipette. The elution procedure was repeated ten times for each replicate, after which the eluted virus and MEM were pipetted to snap cap tubes, placed in a cooler with dry ice, and stored in a freezer at -80°C until being tested. The recovery was done for the three replicates of each treatment simultaneously, and gloves and the plastic covering the workbench were changed between treatments to avoid cross-contamination.

### Virus titration and immunofluorescence staining

#### Titration

PEDV titration was performed using Vero cells in 96-well plates, as Chen et al. (2014) described. PRRSV titration was performed using M-145 cells in 96-well plates [10]. After the culture medium was decanted and cells were washed, cells in 96-well plates were inoculated with 10-fold serially diluted PEDV or PRRSV samples, three replicate wells per dilution. Row A was an undiluted sample, while rows B to H were 10-fold serially diluted in post-inoculation media. After 1 hour of incubation at 37°C with 5% CO_2_, the inoculum was removed, and new post-inoculation media was added for PEDV and PRRSV, respectively. The plates were then placed back in the incubator at 37°C with 5% CO_2_ for five days. The development of the cytopathic effect was monitored once daily. Immunofluorescence staining was conducted as described below.

#### Immunofluorescence staining

Vero cell plates with PEDV and M-145 cell plates with PRRSV samples were incubated for five days at 37°C with 5% CO2. All media was removed from the plates; the cells were washed and then fixed with cold 80% acetone for 10 minutes. Fixed cell plates for PEDV were air-dried and then stained with 50 μL/well of 100x dilution of FITC-conjugated PEDV nucleocapsid protein-specific monoclonal antibody SD-1F-1 (Medgene Labs; Brookings, South Dakota). Fixed cell plates for PRRSV were air-dried and then stained with 50 μL/well of 100x dilution of FITC-conjugated PRRSV nucleocapsid protein-specific monoclonal antibody SDOW17-F (RTI LLC; Brookings, South Dakota). After being held for 1 hour with the antibody conjugate, cells were washed three times with PBS, 5 min per time. The immunofluorescence staining was examined under a fluorescence microscope. The Reed-Muench method was used to calculate the end-point titer. The limit of detection of this titration assay was 1.78 x 10 log_10_TCID_50_/ml. The PRRSV and PEDV titration results are available in S1, S2 Tables respectively, in the supporting information.

### Statistical analysis

The data was divided by surface and temperature and linear regression models using the log_10_ TCID_50_/ml as the outcome and holding-time as a predictor were built. Statistical significance of the holding-time was assessed using analysis of variance (ANOVA) and differences between levels of holding-time were assessed using the Sidak method; results were reported using the marginal means (least-square means). The time to not detect the virus was then estimated for each temperature and surface combination by observing the time the marginal means reached 0 log_10_TCID_50_/ml. The half-lives, using results from the regression models, were calculated for each temperature, surface, and virus using Excel, according to [19]. An equation using the natural logarithm of the estimated marginal means (X) over the estimated marginal means after 15 minutes (Y) was implemented to calculate the half-lives. A regression model was applied using the result from the natural logarithm as the dependent variable and time as the independent variable, using the following formula: *ln* (*X/Y*) *~ time*

Where: *ln* is the natural logarithm, *X* is the estimated marginal means after 1, 6, 12, 24, and 36 hours, *Y* is the estimated marginal means after 15 minutes.

The exponential decay constant was divided by the slope resultant from the regression model to get the half-life time using the formula: ln (2)/*β*

Where: ln (2) is the exponential decay constant, and *β* is the slope from the regression model.

## Results

### PRRSV 1-4-4 L1C variant

PRRSV 1-4-4 L1C variant was still present on both the aluminum and the cardboard surfaces after 36 hours at 20°C, however, no virus was detected after 12 hours at 30°C, after 6 hours at 40°C, or after 15 min at 50°C on the aluminum surfaces. In contrast, no virus was detected on the cardboard surfaces after 6 hours at 30°C, 40°C, or 50°C.

For the 36-hour holding-time at 20°C, PRRSV 1-4-4 L1C variant titer was reduced by 0.92 log_10_TCID_50_/ml and 2.75 log_10_TCID_50_/ml on aluminum and cardboard, respectively. At 30°C, the virus titer was reduced by 3.50 log_10_TCID_50_/ml after 12 hours for aluminum and 3.08 log_10_TCID_50_/ml after 6 hours for cardboard. After 6 hours, the virus titer at 40°C was reduced by 4.08 log_10_TCID_50_/ml on aluminum and by 2.00 log_10_TCID_50_/ml on cardboard. Finally, at 50°C, the virus titer was reduced by 3.33 log_10_TCID_50_/ml after 6 hours on cardboard and was not detected after 15 minutes on aluminum (Table 1).

**Table 1:** Summary of results.

Virus	Temperature	Surface	log10 after 15 minutes	log10 after 1 hour	log10 after 6 hours	log10 after 12 hours	log10 after 24 hours	log10 after 36 hours	No virus detection	Half-life
**PRRSV 1-4-4 L1C Variant**	20°C	Aluminum	3.50 ^bc^	3.94 ^c^	3.25 ^ab^	3.33 ^bc^	2.58^a^	2.58^a^	Virus detected	63 hr
	30°C		3.50 ^c^	2.83 ^bc^	2.47^b^	0.00 ^a^	0.00 ^a^	0.00 ^a^	12 hours	2 hr
	40°C		4.08 ^c^	2.58 ^b^	0.00 ^a^	0.00 ^a^	0.00 ^a^	0.00 ^a^	6 hours	4 hr
	50°C		0.00 ^a^	0.00 ^a^	0.00 ^a^	0.00 ^a^	0.00 ^a^	0.00 ^a^	15 minutes	0 min
	20°C	Cardboard	3.17^b^	2.81^b^	0.50 ^a^	1.83 ^ab^	1.58 ^ab^	0.42 ^a^	Virus detected	18 hr
	30°C		3.08 ^b^	1.42 ^a^	0.00 ^a^	0.00 ^a^	0.00 ^a^	0.00 ^a^	6 hours	5 hr
	40°C		2.00 ^a^	1.33 ^a^	0.00 ^a^	0.00 ^a^	0.00 ^a^	0.00 ^a^	6 hours	47 min
	50°C		3.33 ^b^	1.17 ^a^	0.00 ^a^	0.00 ^a^	0.00 ^a^	0.00 ^a^	6 hours	47 min
**PRRSV MN 184**	20°C	Aluminum	4.50 ^b^	4.33 ^b^	3.64 ^ab^	3.25 ^a^	3.25 ^a^	3.17 ^a^	Virus detected	76 hr
	30°C		4.25 ^c^	3.58 ^c^	2.92 ^bc^	1.42 ^ab^	0.33 ^a^	0.00 ^a^	36 hours	50 min
	40°C		4.25 ^c^	3.65 ^c^	2.06 ^b^	1.42 ^a^	0.00 ^a^	0.00 ^a^	24 hours	16 min
	50°C		3.92 ^c^	1.83 ^b^	0.00 ^a^	0.00 ^a^	0.00 ^a^	0.00 ^a^	6 hours	21 min
	20°C	Cardboard	3.75 ^c^	3.58 ^bc^	2.83 ^ab^	3.08 ^abc^	2.67 ^a^	2.67 ^a^	Virus detected	81 hr
	30°C		4.17 ^b^	3.75 ^b^	2.92 ^b^	0.92 ^a^	2.67 ^a^	0.00 ^a^	36 hours	50 min
	40°C		4.75 ^c^	3.82 ^c^	2.72 ^b^	0.00 ^a^	0.00 ^a^	0.00 ^a^	12 hours	20 min
	50°C		3.81 ^c^	3.83 ^c^	2.83 ^bc^	0.00 ^a^	0.00 ^a^	0.00 ^a^	12 hours	16 min
**PEDV**	20°C	Aluminum	5.50 ^c^	4.83 ^c^	3.92 ^bc^	3.00 ^b^	2.67 ^b^	0.83 ^a^	Virus detected	15 hr
	30°C		3.58 ^c^	2.17 ^b^	0.00 ^a^	0.00 ^a^	0.00 ^a^	0.00 ^a^	6 hours	47 min
	40°C		2.83 ^b^	2.75 ^b^	0.00 ^a^	0.00 ^a^	0.00 ^a^	0.00 ^a^	6 hours	46 min
	50°C		0.00 ^a^	0.00 ^a^	0.00 ^a^	0.00 ^a^	0.00 ^a^	0.00 ^a^	15 minutes	0 min
	20°C	Cardboard	4.58 ^b^	4.58 ^b^	3.83 ^b^	1.83 ^a^	1.92 ^a^	1.83 ^a^	Virus detected	25 hr 30 min
	30°C		3.50 ^b^	2.31 ^b^	0.58 ^a^	0.00 ^a^	0.00 ^a^	0.00 ^a^	12 hours	36 min
	40°C		3.33 ^c^	2.75 ^bc^	1.00 ^ab^	0.00 ^a^	0.00 ^a^	0.00 ^a^	12 hours	52 min
	50°C		3.42 ^c^	1.58 ^b^	0.50 ^ab^	0.00 ^a^	0.00 ^a^	0.00 ^a^	12 hours	36 min

Summary of the marginal means of the log_10_TCID_50_/ml values for each virus, surface and holding-time, and summary of the time to virus detection.

**Fig 2:**
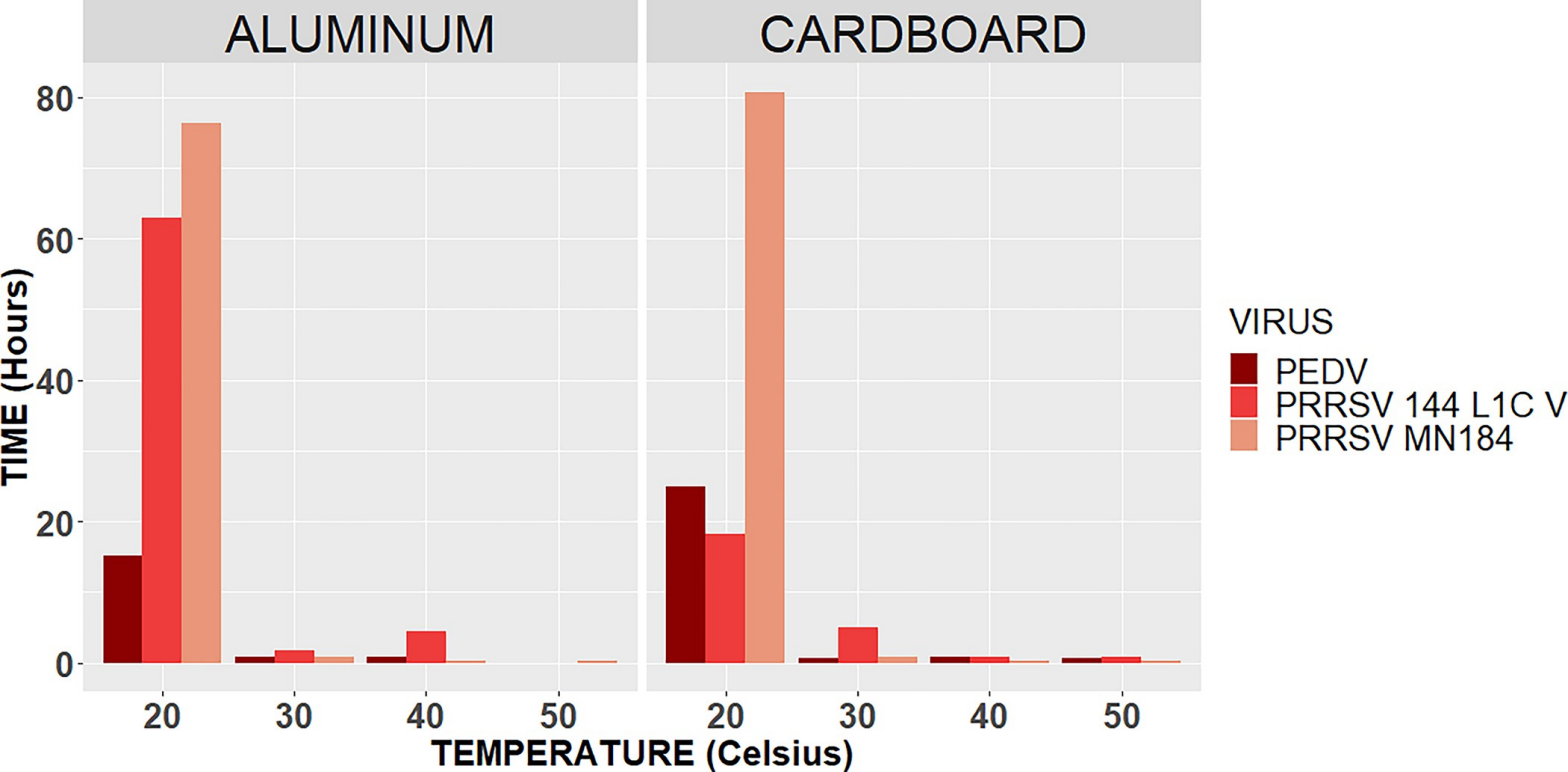
Half-lives per surface. Representation of the half-life in hours for each virus (PEDV, PRRSV 1-4-4 L1C variant, and PRRSV MN 184) by each surface type (aluminum and cardboard).

The half-lives of the PRRSV 144 L1C variant at 30°C were 5 hours for cardboard and 2 hours for aluminum. At 40°C, the half-lives were 4 hours on aluminum and 47 minutes on cardboard; at 50°C, the half-lives were 0 minutes on aluminum and 47 minutes on cardboard (Fig 2).

### PRRSV MN 184

Both surfaces had the live virus after 36 hours at 20°C. At 30°C, no virus was detected after 36 hours on aluminum and was not detected on cardboard after 24 hours, although one of the three replicates after 36 hours had 10^1^ log_10_TCID_50_/ml. Since no virus was present after 24 hours and only one of the three replicates turned out positive, a negative result after 36 hours was assumed for the analysis. No virus was detected on the aluminum surface after 24 hours at 40°C and 6 hours at 50°C. On the cardboard surface, no virus was detected after 12 hours at either 40°C or 50°C.

During the 36 hours holding-time at 20°C, PRRSV MN 184 titer was reduced by 1.33 log_10_TCID_50_/ml and 1.08 log on aluminum and cardboard, respectively. At 30°C, the virus titer was reduced by 4.25 log_10_TCID_50_/ml after 36 hours on the aluminum and by 4.17 log_10_TCID_50_/ml after 36 hours on cardboard. At 40°C, after 24 hours, the virus titer was reduced by 4.25 log10TCID50/ml on aluminum; after 12 hours, the virus titer was reduced by 4.75 log_10_TCID_50_/ml on cardboard. Finally, at 50°C, the virus titer was reduced by 3.92 log_10_TCID_50_/ml after 6 hours on aluminum and by 3.81 log_10_TCID_50_/ml after 12 hours on cardboard (Table 1).

Half-lives of PRRSV MN184 at 30°C were 50 minutes on both surfaces. At 40°C, the half-lives were 16 minutes on aluminum and 20 minutes on cardboard. Finally, at 50°C, the half-lives were 21 minutes on aluminum and 16 minutes on cardboard (Fig 2).

#### PEDV

The virus was present after 36 hours at 20°C on both the aluminum and the cardboard surfaces. No live virus was detected on the aluminum at 30°C and 40°C after 6 hours and at 50°C after 15 min. No live virus was detected on the cardboard after 12 hours at 30°C, 40°C, and 50°C.

During the 36 hours holding-time at 20°C, PEDV titer was reduced by 4.67 log_10_TCID_50_/ml and 2.75 log_10_TCID_50_/ml on the aluminum and the cardboard, respectively. At 30°C, the virus titer was reduced by 3.58 log_10_TCID_50_/ml after 6 hours on the aluminum and by 3.50 log_10_TCID_50_/ml after 12 hours on the cardboard. At 40°C, after 6 hours, the virus titer was reduced by 2.83 log_10_TCID_50_/ml on the aluminum, and after 12 hours, the virus titer was reduced by 3.33 log_10_TCID_50_/ml on the cardboard. Finally, at 50°C, the virus titer was reduced by 3.42 log_10_TCID_50_/ml after 12 hours on the cardboard and was already 0.00 log_10_TCID_50_/ml after 15 minutes on the aluminum (Table 1).

At 30°C, the half-lives were 36 minutes on cardboard and 47 minutes on aluminum, at 40°C the half-lives were 46 minutes on aluminum and 52 minutes on cardboard, and at 50°C the half-lives were 0 minutes on aluminum and 36 minutes on cardboard (Fig 2).

**Fig 3:**
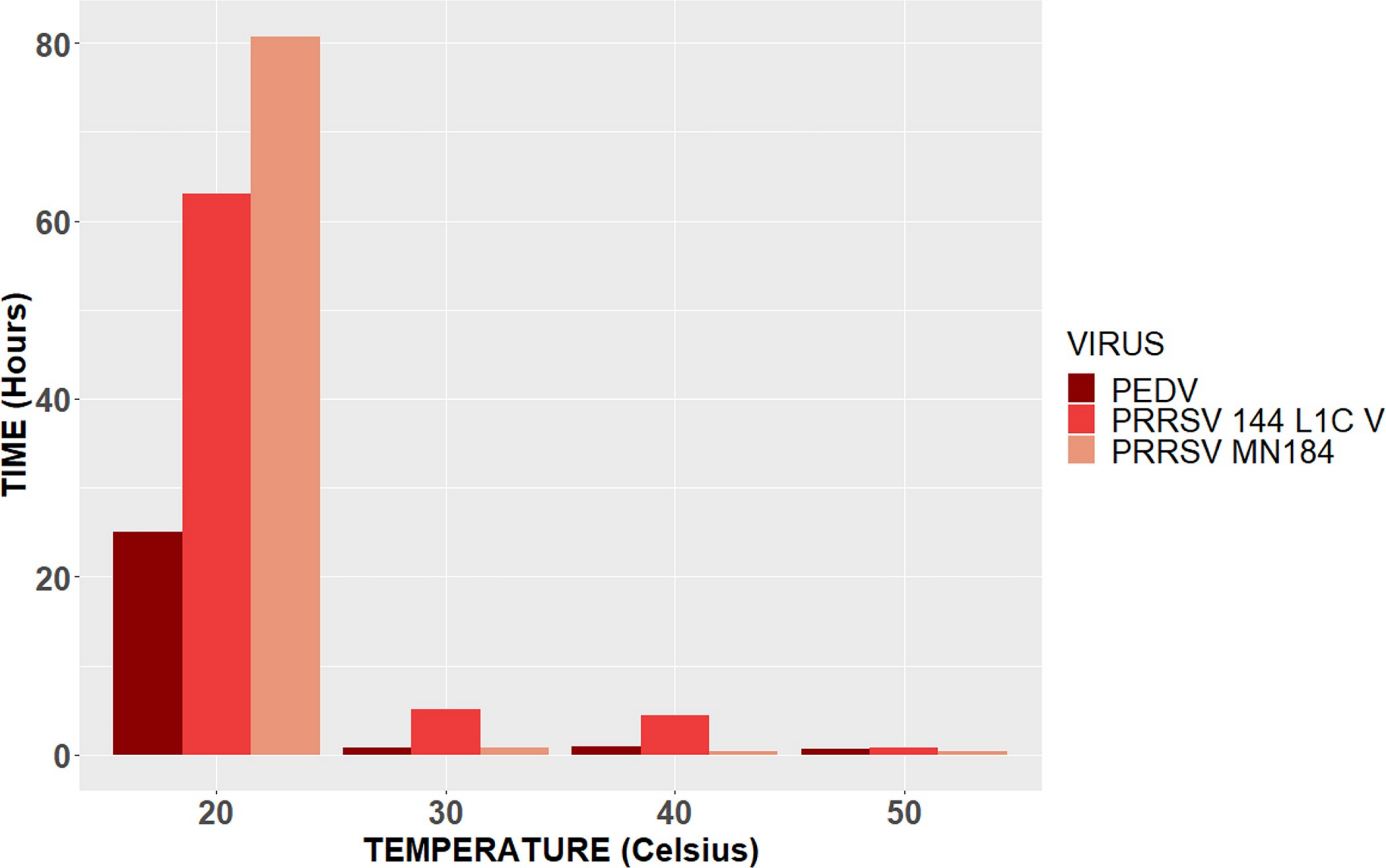
Overall half-lives. Representation of the overall half-life in hours for PEDV, PRRSV 1-4-4 L1C variant, and PRRSV MN 184.

A comparison between all viruses and their half-lives can be observed in Fig 3.

## Discussion

Applying temperature and time combinations to decrease the probability of virus introduction is feasible under field conditions. For this study, several experts (swine practitioners, researchers, and virologists) agreed with the selection of four temperatures and six holding times that can be easily replicated in supply entry rooms. Heat is an alternative to disinfectants because heat is conducted through the material, porous or not, reaching every surface and the content of boxes, dehydrating organic matter and decreasing the probability of virus detection.

The half-life for PRRSV MN 184 at 20°C was 81 hours on cardboard and 76 hours on aluminum. For PRRSV 1-4-4 L1C variant was 18 hours on cardboard and 63 hours on aluminum. At 30°C, the half-life for PRRSV MN 184 was 50 minutes on both surfaces, and for PRRSV 1-4-4 L1C variant was 5 hours on cardboard and 2 hours on aluminum. According to [20], PRRSV half-life in the serum of a viremic pig did not differ between different isolates (ATCC VR-2332, JA142, MN-184, Ingelvac H PRRS ATP vaccine virus [Boehringer Ingelheim Vetmedica Inc., St. Joseph, MO]). Half-lives were estimated to be 84.5 hours at 10°C, 27.4 hours at 20°C, and 1.6 hours at 30°C. In the present study, the half-life results suggest that there seems to be a difference between PRRSV isolates because PRRSV 1-4-4 L1C variant had higher half-lives at higher temperatures than PRRSV MN 184.

[6] showed that PEDV inactivation was possible after seven days at 20°C. This study observed that PEDV was still present after 36 hours at 20°C, although a decrease in log_10_TCID_50_/ml values was observed and reached a value of zero after holding the aluminum surface at 30°C for 6 hours and after holding the cardboard surface at 30°C for 24 hours. [5] compared the concentration of viable PEDV in stainless steel, aluminum, plastic, and galvanized steel. The study showed a log_10_TCID_50_/ml reduction from 3.51 to 2.51 log_10_TCID_50_/ml on an aluminum surface in the first 24 hours of the trial. This study observed a 2.83 log10TCID50/ml value decrease at 20°C after 24 hours on aluminum. In [5], the log_10_TCID_50_/ml value was 2.18 after ten days, emphasizing the extended period of time required to eliminate viruses at 20°C, making it an impractical option for a producer in a swine farm. After periods of 15 minutes and 60 minutes, there was only evidence of PEDV and PRRSV 1-4-4 L1C variant inactivation at 50°C in aluminum surfaces.

Regarding the difference between surface types, it was observed that the aluminum surface took more time to reach the desired temperature when compared to cardboard. This difference may be due to the thermal conductivity of aluminum, that is, the ability to conduct heat [21]. For laminated cardboard, the conductivity is 0.72 W/mK as compared to 160 W/mk for aluminum [22]. Therefore, in field conditions, it is advisable to let aluminum surfaces stay for longer periods in the supply entry room or increase the temperature.

The results from this study can help swine producers to select times and temperatures, according to the conditions at swine farms. It is noteworthy that the treatments were placed in an incubator, which can be smaller compared to supply entry rooms, making heat dispersion different from a regular supply entry room, as well as the time that it takes for different surfaces to reach the desired temperature, due to differences in materials or the dimension of surfaces. Although these combinations of time and temperature can be readily applied to the conditions at swine farms, surfaces with organic matter were not tested, which is an important risk factor in disease introduction [1]. The results from this study should be extrapolated carefully to field conditions that usually have less control compared to this study setting. Time and temperature combinations should not be used for biological materials such as vaccines [23] and need to consider the different characteristics of materials. In addition, when applying time and temperature treatment in field conditions, the room insolation, size, and the number of materials should be considered. Overall, each farm is different, in terms of facilities, biosecurity protocols, materials, and health statuses, and all of those should be considered when implementing this type of combination of time and temperature to get a successful decrease in virus concentration.

Another aspect that should be considered is the assay used to determine virus viability at the end of each treatment. For instance, [6] applied a bioassay to determine PEDV viability in 28 treatments of aluminum surfaces with feces and PEDV that were treated with different combinations of time and temperature. For this study, virus viability in treated samples was determined in cell culture and was not confirmed by pig bioassay. However, since there were no organic materials (e.g., feces) or disinfectants in the treatments, cytotoxicity caused by organic materials or disinfectants was not encountered in this study, and the virus titration results are trustworthy.

## Conclusion

In conclusion, under the conditions of this study, it was observed that using combinations of time and temperature was effective in decreasing the concentration of PRRSV and PEDV on different surfaces commonly found in supply entry rooms at swine farms. The results from the marginal means suggest that the minimum time that surfaces should be held to prevent virus detection at 30°C was 24 hours, at 40°C was 12 hours, and at 50°C was 6 hours; aluminum surfaces took longer to reach the desired temperature. According to the half-lives, there seems to be a difference between PRRSV isolates, with PRRSV 1-4-4 L1C variant having longer half-lives at higher temperatures compared to the PRRSV MN 184 isolate.

## Supporting information

S1 Table PRRSV titration results. PRRSV MN 1-8-4 and PRRSV 1-4-4 L1C Variant titration results per surface, contact time and temperature.
(PDF)

S2 Table PEDV titration results. Results of PEDV titrations per surface, contact time and temperature.
(PDF)
