# Supplementary material for: Assessment of temperature and time on the survivability of porcine reproductive and respiratory syndrome virus (PRRSV) and porcine epidemic diarrhea virus (PEDV) on experimentally contaminated surfaces
Source: PLoS One. 2024 Jan 19;19(1):e0291181. doi: 10.1371/journal.pone.0291181 (PMC10798431; doi:10.1371/journal.pone.0291181)
Supplement: S1 Table — PRRSV MN 1-8-4 and PRRSV 1-4-4 L1C Variant titration results per surface, contact time and temperature. (PDF) [file pone.0291181.s001.pdf]

S1 Table. PRRSV titration results.

Study group	Series #	ATCID	BTCID	CTCID	Average TCID	A LOG	B LOG	C LOG	Average LOG	Surface	Contact time (min)	Temperature (ºF)	Temperature (°C)
Negative Control	1 A-C	0.00E+00	0.00E+00	0.00E+00	0.00E+00	0.00	0.00	0.00	0.00	Cardboard	2160	68ºF	20°C
Negative Control	2 A-C	0.00E+00	0.00E+00	0.00E+00	0.00E+00	0.00	0.00	0.00	0.00	Aluminum	2160	68ºF	20°C
PRRSV MN 1-8-4	3 A-C	3.16E+03	3.16E+03	1.78E+04	5.62E+03	3.50	3.50	4.25	3.75	Cardboard	15	68ºF	20°C
PRRSV MN 1-8-4	4 A-C	3.16E+04	3.16E+04	3.16E+04	3.16E+04	4.50	4.50	4.50	4.50	Aluminum	15	68ºF	20°C
PRRSV 1-4-4 L1C V	5 A-C	5.62E+02	3.16E+03	1.78E+03	1.47E+03	2.75	3.50	3.25	3.17	Cardboard	15	68ºF	20°C
PRRSV 1-4-4 L1C V	6 A-C	3.16E+03	3.16E+03	3.16E+03	3.16E+03	3.50	3.50	3.50	3.50	Aluminum	15	68ºF	20°C
PRRSV MN 1-8-4	9 A-C	3.16E+03	3.16E+03	5.62E+03	3.83E+03	3.50	3.50	3.75	3.58	Cardboard	60	68ºF	20°C
PRRSV MN 1-8-4	10 A-C	1.78E+04	1.78E+04	3.16E+04	2.16E+04	4.25	4.25	4.50	4.33	Aluminum	60	68ºF	20°C
PRRSV 1-4-4 L1C V	11 A-C	4.64E+02	1.78E+03	3.16E+02	6.39E+02	2.67	3.25	2.50	2.81	Cardboard	60	68ºF	20°C
PRRSV 1-4-4 L1C V	12 A-C	5.62E+03	6.81E+03	1.78E+04	8.80E+03	3.75	3.83	4.25	3.94	Aluminum	60	68ºF	20°C
PRRSV MN 1-8-4	15 A-C	3.16E+02	1.78E+03	5.62E+02	6.81E+02	2.50	3.25	2.75	2.83	Cardboard	360	68ºF	20°C
PRRSV MN 1-8-4	16 A-C	4.64E+03	3.16E+03	5.62E+03	4.35E+03	3.67	3.50	3.75	3.64	Aluminum	360	68ºF	20°C
PRRSV 1-4-4 L1C V	17 A-C	0.00E+00	3.16E+01	0.00E+00	3.16E+00	0.00	1.50	0.00	0.50	Cardboard	360	68ºF	20°C
PRRSV 1-4-4 L1C V	18 A-C	1.00E+03	1.78E+03	3.16E+03	1.78E+03	3.00	3.25	3.50	3.25	Aluminum	360	68ºF	20°C
PRRSV MN 1-8-4	21 A-C	3.16E+02	1.00E+03	3.16E+02	4.64E+02	2.50	3.00	2.50	2.67	Cardboard	1440	68ºF	20°C
PRRSV MN 1-8-4	22 A-C	1.78E+03	5.62E+02	5.62E+03	1.78E+03	3.25	2.75	3.75	3.25	Aluminum	1440	68ºF	20°C
PRRSV 1-4-4 L1C V	23 A-C	3.16E+01	3.16E+01	5.62E+01	3.83E+01	1.50	1.50	1.75	1.58	Cardboard	1440	68ºF	20°C
PRRSV 1-4-4 L1C V	24 A-C	5.62E+02	1.78E+02	5.62E+02	3.83E+02	2.75	2.25	2.75	2.58	Aluminum	1440	68ºF	20°C
PRRSV MN 1-8-4	27 A-C	1.78E+03	1.78E+03	5.62E+02	1.21E+03	3.25	3.25	2.75	3.08	Cardboard	720	68ºF	20°C
PRRSV MN 1-8-4	28 A-C	5.62E+03	3.16E+03	3.16E+02	1.78E+03	3.75	3.50	2.50	3.25	Aluminum	720	68ºF	20°C

PRRSV 1-4-4 L1C V	29 A-C	3.16E+02	3.16E+01	3.16E+01	6.81E+01	2.50	1.50	1.50	1.83	Cardboard	720	68ºF	20°C
PRRSV 1-4-4 L1C V	30 A-C	1.00E+03	3.16E+03	3.16E+03	2.15E+03	3.00	3.50	3.50	3.33	Aluminum	720	68ºF	20°C
PRRSV MN 1-8-4	33 A-C	5.62E+02	5.62E+02	3.16E+02	4.64E+02	2.75	2.75	2.50	2.67	Cardboard	2160	68ºF	20°C
PRRSV MN 1-8-4	34 A-C	5.62E+02	1.78E+03	3.16E+03	1.47E+03	2.75	3.25	3.50	3.17	Aluminum	2160	68ºF	20°C
PRRSV 1-4-4 L1C V	35 A-C	0.00E+00	1.78E+01	0.00E+00	2.61E+00	0.00	1.25	0.00	0.42	Cardboard	2160	68ºF	20°C
PRRSV 1-4-4 L1C V	36 A-C	3.16E+02	3.16E+02	5.62E+02	3.83E+02	2.50	2.50	2.75	2.58	Aluminum	2160	68ºF	20°C
PRRSV MN 1-8-4	39 A-C	1.78E+04	3.16E+04	5.62E+03	1.47E+04	4.25	4.50	3.75	4.17	Cardboard	15	86ºF	30°C
PRRSV MN 1-8-4	40 A-C	1.78E+04	5.62E+04	5.62E+03	1.78E+04	4.25	4.75	3.75	4.25	Aluminum	15	86ºF	30°C
PRRSV 1-4-4 L1C V	41 A-C	3.16E+02	1.78E+03	3.16E+03	1.21E+03	2.50	3.25	3.50	3.08	Cardboard	15	86ºF	30°C
PRRSV 1-4-4 L1C V	42 A-C	3.16E+03	5.62E+03	1.78E+03	3.16E+03	3.50	3.75	3.25	3.50	Aluminum	15	86ºF	30°C
PRRSV MN 1-8-4	45 A-C	1.78E+04	3.16E+03	3.16E+03	5.62E+03	4.25	3.50	3.50	3.75	Cardboard	60	86ºF	30°C
PRRSV MN 1-8-4	46 A-C	3.16E+03	3.16E+03	5.62E+03	3.83E+03	3.50	3.50	3.75	3.58	Aluminum	60	86ºF	30°C
PRRSV 1-4-4 L1C V	47 A-C	0.00E+00	1.00E+02	1.78E+02	2.61E+01	0.00	2.00	2.25	1.42	Cardboard	60	86ºF	30°C
PRRSV 1-4-4 L1C V	48 A-C	3.16E+02	3.16E+02	3.16E+03	6.81E+02	2.50	2.50	3.50	2.83	Aluminum	60	86ºF	30°C
PRRSV MN 1-8-4	51 A-C	5.62E+02	3.16E+03	3.16E+02	8.25E+02	2.75	3.50	2.50	2.92	Cardboard	360	86ºF	30°C
PRRSV MN 1-8-4	52 A-C	5.62E+02	5.62E+02	1.78E+03	8.25E+02	2.75	2.75	3.25	2.92	Aluminum	360	86ºF	30°C
PRRSV 1-4-4 L1C V	53 A-C	0.00E+00	0.00E+00	0.00E+00	0.00E+00	0.00	0.00	0.00	0.00	Cardboard	360	86ºF	30°C
PRRSV 1-4-4 L1C V	54 A-C	4.46E+02	1.78E+02	3.16E+02	2.93E+02	2.65	2.25	2.50	2.47	Aluminum	360	86ºF	30°C
PRRSV MN 1-8-4	57 A-C	0.00E+00	0.00E+00	0.00E+00	0.00E+00	0.00	0.00	0.00	0.00	Cardboard	1440	86ºF	30°C
PRRSV MN 1-8-4	58 A-C	0.00E+00	0.00E+00	1.00E+01	2.15E+00	0.00	0.00	1.00	0.33	Aluminum	1440	86ºF	30°C
PRRSV 1-4-4 L1C V	59 A-C	0.00E+00	0.00E+00	0.00E+00	0.00E+00	0.00	0.00	0.00	0.00	Cardboard	1440	86ºF	30°C
PRRSV 1-4-4 L1C V	60 A-C	0.00E+00	0.00E+00	0.00E+00	0.00E+00	0.00	0.00	0.00	0.00	Aluminum	1440	86ºF	30°C
PRRSV MN 1-8-4	63 A-C	0.00E+00	3.16E+01	1.78E+01	8.25E+00	0.00	1.50	1.25	0.92	Cardboard	720	86ºF	30°C

PRRSV MN 1-8-4	64 A-C	0.00E+00	3.16E+01	5.62E+02	2.61E+01	0.00	1.50	2.75	1.42	Aluminum	720	86ºF	30°C
PRRSV 1-4-4 L1C V	65 A-C	0.00E+00	0.00E+00	0.00E+00	0.00E+00	0.00	0.00	0.00	0.00	Cardboard	720	86ºF	30°C
PRRSV 1-4-4 L1C V	66 A-C	0.00E+00	0.00E+00	0.00E+00	0.00E+00	0.00	0.00	0.00	0.00	Aluminum	720	86ºF	30°C
PRRSV MN 1-8-4	69 A-C	0.00E+00	0.00E+00	1.00E+01	2.15E+00	0.00	0.00	1.00	0.33	Cardboard	2160	86ºF	30°C
PRRSV MN 1-8-4	70 A-C	0.00E+00	0.00E+00	0.00E+00	0.00E+00	0.00	0.00	0.00	0.00	Aluminum	2160	86ºF	30°C
PRRSV 1-4-4 L1C V	71 A-C	0.00E+00	0.00E+00	0.00E+00	0.00E+00	0.00	0.00	0.00	0.00	Cardboard	2160	86ºF	30°C
PRRSV 1-4-4 L1C V	72 A-C	0.00E+00	0.00E+00	0.00E+00	0.00E+00	0.00	0.00	0.00	0.00	Aluminum	2160	86ºF	30°C
PRRSV MN 1-8-4	75 A-C	3.16E+05	3.16E+04	1.78E+04	5.62E+04	5.50	4.50	4.25	4.75	Cardboard	15	104ºF	40°C
PRRSV MN 1-8-4	76 A-C	1.78E+04	1.78E+04	1.78E+04	1.78E+04	4.25	4.25	4.25	4.25	Aluminum	15	104ºF	40°C
PRRSV 1-4-4 L1C V	77 A-C	5.62E+02	1.78E+03	0.00E+00	1.00E+02	2.75	3.25	0.00	2.00	Cardboard	15	104ºF	40°C
PRRSV 1-4-4 L1C V	78 A-C	3.16E+04	1.78E+04	3.16E+03	1.21E+04	4.50	4.25	3.50	4.08	Aluminum	15	104ºF	40°C
PRRSV MN 1-8-4	81 A-C	3.16E+03	2.85E+04	3.16E+03	6.58E+03	3.50	4.45	3.50	3.82	Cardboard	60	104ºF	40°C
PRRSV MN 1-8-4	82 A-C	1.00E+04	3.16E+03	2.85E+03	4.48E+03	4.00	3.50	3.45	3.65	Aluminum	60	104ºF	40°C
PRRSV 1-4-4 L1C V	83 A-C	0.00E+00	5.62E+01	1.78E+02	2.15E+01	0.00	1.75	2.25	1.33	Cardboard	60	104ºF	40°C
PRRSV 1-4-4 L1C V	84 A-C	5.62E+02	1.78E+02	5.62E+02	3.83E+02	2.75	2.25	2.75	2.58	Aluminum	60	104ºF	40°C
PRRSV MN 1-8-4	87 A-C	4.64E+02	5.62E+02	5.62E+02	5.27E+02	2.67	2.75	2.75	2.72	Cardboard	360	104ºF	40°C
PRRSV MN 1-8-4	88 A-C	4.64E+01	5.62E+01	5.62E+02	1.14E+02	1.67	1.75	2.75	2.06	Aluminum	360	104ºF	40°C
PRRSV 1-4-4 L1C V	89 A-C	0.00E+00	0.00E+00	0.00E+00	0.00E+00	0.00	0.00	0.00	0.00	Cardboard	360	104ºF	40°C
PRRSV 1-4-4 L1C V	90 A-C	0.00E+00	0.00E+00	0.00E+00	0.00E+00	0.00	0.00	0.00	0.00	Aluminum	360	104ºF	40°C
PRRSV MN 1-8-4	93 A-C	0.00E+00	0.00E+00	0.00E+00	0.00E+00	0.00	0.00	0.00	0.00	Cardboard	1440	104ºF	40°C
PRRSV MN 1-8-4	94 A-C	0.00E+00	0.00E+00	0.00E+00	0.00E+00	0.00	0.00	0.00	0.00	Aluminum	1440	104ºF	40°C
PRRSV 1-4-4 L1C V	95 A-C	0.00E+00	0.00E+00	0.00E+00	0.00E+00	0.00	0.00	0.00	0.00	Cardboard	1440	104ºF	40°C
PRRSV 1-4-4 L1C V	96 A-C	0.00E+00	0.00E+00	0.00E+00	0.00E+00	0.00	0.00	0.00	0.00	Aluminum	1440	104ºF	40°C

PRRSV MN 1-8-4	99 A-C	0.00E+00	0.00E+00	0.00E+00	0.00E+00	0.00	0.00	0.00	0.00	Cardboard	720	104ºF	40°C
PRRSV MN 1-8-4	100 A-C	0.00E+00	0.00E+00	0.00E+00	0.00E+00	0.00	0.00	0.00	0.00	Aluminum	720	104ºF	40°C
PRRSV 1-4-4 L1C V	101 A-C	0.00E+00	0.00E+00	0.00E+00	0.00E+00	0.00	0.00	0.00	0.00	Cardboard	720	104ºF	40°C
PRRSV 1-4-4 L1C V	102 A-C	0.00E+00	0.00E+00	0.00E+00	0.00E+00	0.00	0.00	0.00	0.00	Aluminum	720	104ºF	40°C
PRRSV MN 1-8-4	105 A-C	0.00E+00	0.00E+00	0.00E+00	0.00E+00	0.00	0.00	0.00	0.00	Cardboard	2160	104ºF	40°C
PRRSV MN 1-8-4	106 A-C	0.00E+00	0.00E+00	0.00E+00	0.00E+00	0.00	0.00	0.00	0.00	Aluminum	2160	104ºF	40°C
PRRSV 1-4-4 L1C V	107 A-C	0.00E+00	0.00E+00	0.00E+00	0.00E+00	0.00	0.00	0.00	0.00	Cardboard	2160	104ºF	40°C
PRRSV 1-4-4 L1C V	108 A-C	0.00E+00	0.00E+00	0.00E+00	0.00E+00	0.00	0.00	0.00	0.00	Aluminum	2160	104ºF	40°C
PRRSV MN 1-8-4	111 A-C	3.16E+03	1.78E+04	4.64E+03	6.39E+03	3.50	4.25	3.67	3.81	Cardboard	15	122ºF	50°C
PRRSV MN 1-8-4	112 A-C	3.16E+03	3.16E+04	5.62E+03	8.25E+03	3.50	4.50	3.75	3.92	Aluminum	15	122ºF	50°C
PRRSV 1-4-4 L1C V	113 A-C	5.62E+03	3.16E+03	5.62E+02	2.15E+03	3.75	3.50	2.75	3.33	Cardboard	15	122ºF	50°C
PRRSV 1-4-4 L1C V	114 A-C	0.00E+00	0.00E+00	0.00E+00	0.00E+00	0.00	0.00	0.00	0.00	Aluminum	15	122ºF	50°C
PRRSV MN 1-8-4	117 A-C	1.78E+03	5.62E+03	3.16E+04	6.81E+03	3.25	3.75	4.50	3.83	Cardboard	60	122ºF	50°C
PRRSV MN 1-8-4	118 A-C	3.16E+01	3.16E+02	3.16E+01	6.81E+01	1.50	2.50	1.50	1.83	Aluminum	60	122ºF	50°C
PRRSV 1-4-4 L1C V	119 A-C	3.16E+01	0.00E+00	1.00E+02	1.47E+01	1.50	0.00	2.00	1.17	Cardboard	60	122ºF	50°C
PRRSV 1-4-4 L1C V	120 A-C	0.00E+00	0.00E+00	0.00E+00	0.00E+00	0.00	0.00	0.00	0.00	Aluminum	60	122ºF	50°C
PRRSV MN 1-8-4	123 A-C	5.62E+02	3.16E+02	1.78E+03	6.81E+02	2.75	2.50	3.25	2.83	Cardboard	360	122ºF	50°C
PRRSV MN 1-8-4	124 A-C	0.00E+00	0.00E+00	0.00E+00	0.00E+00	0.00	0.00	0.00	0.00	Aluminum	360	122ºF	50°C
PRRSV 1-4-4 L1C V	125 A-C	0.00E+00	0.00E+00	0.00E+00	0.00E+00	0.00	0.00	0.00	0.00	Cardboard	360	122ºF	50°C
PRRSV 1-4-4 L1C V	126 A-C	0.00E+00	0.00E+00	0.00E+00	0.00E+00	0.00	0.00	0.00	0.00	Aluminum	360	122ºF	50°C
PRRSV MN 1-8-4	129 A-C	0.00E+00	0.00E+00	0.00E+00	0.00E+00	0.00	0.00	0.00	0.00	Cardboard	1440	122ºF	50°C
PRRSV MN 1-8-4	130 A-C	0.00E+00	0.00E+00	0.00E+00	0.00E+00	0.00	0.00	0.00	0.00	Aluminum	1440	122ºF	50°C
PRRSV 1-4-4 L1C V	131 A-C	0.00E+00	0.00E+00	0.00E+00	0.00E+00	0.00	0.00	0.00	0.00	Cardboard	1440	122ºF	50°C

PRRSV 1-4-4 L1C V	132 A-C	0.00E+00	0.00E+00	0.00E+00	0.00E+00	0.00	0.00	0.00	0.00	Aluminum	1440	122ºF	50°C
PRRSV MN 1-8-4	135 A-C	0.00E+00	0.00E+00	0.00E+00	0.00E+00	0.00	0.00	0.00	0.00	Cardboard	720	122ºF	50°C
PRRSV MN 1-8-4	136 A-C	0.00E+00	0.00E+00	0.00E+00	0.00E+00	0.00	0.00	0.00	0.00	Aluminum	720	122ºF	50°C
PRRSV 1-4-4 L1C V	137 A-C	0.00E+00	0.00E+00	0.00E+00	0.00E+00	0.00	0.00	0.00	0.00	Cardboard	720	122ºF	50°C
PRRSV 1-4-4 L1C V	138 A-C	0.00E+00	0.00E+00	0.00E+00	0.00E+00	0.00	0.00	0.00	0.00	Aluminum	720	122ºF	50°C
PRRSV MN 1-8-4	141 A-C	0.00E+00	0.00E+00	0.00E+00	0.00E+00	0.00	0.00	0.00	0.00	Cardboard	2160	122ºF	50°C
PRRSV MN 1-8-4	142 A-C	0.00E+00	0.00E+00	0.00E+00	0.00E+00	0.00	0.00	0.00	0.00	Aluminum	2160	122ºF	50°C
PRRSV 1-4-4 L1C V	143 A-C	0.00E+00	0.00E+00	0.00E+00	0.00E+00	0.00	0.00	0.00	0.00	Cardboard	2160	122ºF	50°C
PRRSV 1-4-4 L1C V	144 A-C	0.00E+00	0.00E+00	0.00E+00	0.00E+00	0.00	0.00	0.00	0.00	Aluminum	2160	122ºF	50°C

PRRSV MN 1-8-4 and PRRSV 1-4-4 L1C Variant titration results per surface, contact time and temperature.
