# Supplementary material for: Assessment of temperature and time on the survivability of porcine reproductive and respiratory syndrome virus (PRRSV) and porcine epidemic diarrhea virus (PEDV) on experimentally contaminated surfaces
Source: PLoS One. 2024 Jan 19;19(1):e0291181. doi: 10.1371/journal.pone.0291181 (PMC10798431; doi:10.1371/journal.pone.0291181)
Supplement: S2 Table — Results of PEDV titrations per surface, contact time and temperature. (PDF) [file pone.0291181.s002.pdf]

S2 Table. PEDV titration results.

Study group	Series #	ATCID	BTCID	CTCID	Average TCID	A LOG	B LOG	C LOG	Average LOG	Surface	Contact time (min)	Temperature (°F)	Temperature (°C)
Negative Control	1 A-C	0.00E+00	0.00E+00	0.00E+00	0.00E+00	0.00	0.00	0.00	0.00	Cardboard	2160	68°F	20°C
Negative Control	2 A-C	0.00E+00	0.00E+00	0.00E+00	0.00E+00	0.00	0.00	0.00	0.00	Aluminum	2160	68°F	20°C
PEDV	7 A-C	3.16E+04	3.16E+04	5.62E+04	3.83E+04	4.50	4.50	4.75	4.58	Cardboard	15	68°F	20°C
PEDV	8 A-C	1.00E+06	1.00E+05	3.16E+05	3.16E+05	6.00	5.00	5.50	5.50	Aluminum	15	68°F	20°C
PEDV	13 A-C	1.78E+04	1.78E+04	1.78E+05	3.83E+04	4.25	4.25	5.25	4.58	Cardboard	60	68°F	20°C
PEDV	14 A-C	1.78E+05	5.62E+04	3.16E+04	6.81E+04	5.25	4.75	4.50	4.83	Aluminum	60	68°F	20°C
PEDV	19 A-C	1.00E+04	1.00E+04	3.16E+03	6.81E+03	4.00	4.00	3.50	3.83	Cardboard	360	68°F	20°C
PEDV	20 A-C	1.78E+03	1.78E+04	1.78E+04	8.26E+03	3.25	4.25	4.25	3.92	Aluminum	360	68°F	20°C
PEDV	25 A-C	5.62E+01	3.16E+02	3.16E+01	8.25E+01	1.75	2.50	1.50	1.92	Cardboard	1440	68°F	20°C
PEDV	26 A-C	3.16E+02	5.62E+02	5.62E+02	4.64E+02	2.50	2.75	2.75	2.67	Aluminum	1440	68°F	20°C
PEDV	31 A-C	5.62E+01	3.16E+01	1.78E+02	6.81E+01	1.75	1.50	2.25	1.83	Cardboard	720	68°F	20°C
PEDV	32 A-C	5.62E+02	3.16E+02	5.62E+03	9.99E+02	2.75	2.50	3.75	3.00	Aluminum	720	68°F	20°C
PEDV	37 A-C	3.16E+01	3.16E+01	3.16E+02	6.81E+01	1.50	1.50	2.50	1.83	Cardboard	2160	68°F	20°C
PEDV	38 A-C	3.16E+01	1.00E+01	0.00E+00	6.81E+00	1.50	1.00	0.00	0.83	Aluminum	2160	68°F	20°C
PEDV	43 A-C	1.78E+03	5.62E+03	3.16E+03	3.16E+03	3.25	3.75	3.50	3.50	Cardboard	15	86°F	30°C
PEDV	44 A-C	3.16E+03	5.62E+03	3.16E+03	3.83E+03	3.50	3.75	3.50	3.58	Aluminum	15	86°F	30°C
PEDV	49 A-C	3.16E+02	5.62E+01	4.64E+02	2.02E+02	2.50	1.75	2.67	2.31	Cardboard	60	86°F	30°C
PEDV	50 A-C	1.78E+02	5.62E+01	3.16E+02	1.47E+02	2.25	1.75	2.50	2.17	Aluminum	60	86°F	30°C
PEDV	55 A-C	0.00E+00	0.00E+00	5.62E+01	3.83E+00	0.00	0.00	1.75	0.58	Cardboard	360	86°F	30°C
PEDV	56 A-C	0.00E+00	0.00E+00	0.00E+00	0.00E+00	0.00	0.00	0.00	0.00	Aluminum	360	86°F	30°C
PEDV	61 A-C	0.00E+00	0.00E+00	0.00E+00	0.00E+00	0.00	0.00	0.00	0.00	Cardboard	1440	86°F	30°C

PEDV	62 A-C	0.00E+00	0.00E+00	0.00E+00	0.00E+00	0.00	0.00	0.00	0.00	Aluminum	1440	86ºF	30°C
PEDV	67 A-C	0.00E+00	0.00E+00	0.00E+00	0.00E+00	0.00	0.00	0.00	0.00	Cardboard	720	86ºF	30°C
PEDV	68 A-C	0.00E+00	0.00E+00	0.00E+00	0.00E+00	0.00	0.00	0.00	0.00	Aluminum	720	86ºF	30°C
PEDV	73 A-C	0.00E+00	0.00E+00	0.00E+00	0.00E+00	0.00	0.00	0.00	0.00	Cardboard	2160	86ºF	30°C
PEDV	74 A-C	0.00E+00	0.00E+00	0.00E+00	0.00E+00	0.00	0.00	0.00	0.00	Aluminum	2160	86ºF	30°C
PEDV	79 A-C	5.62E+03	5.62E+02	3.16E+03	2.15E+03	3.75	2.75	3.50	3.33	Cardboard	15	104ºF	40°C
PEDV	80 A-C	3.16E+03	3.16E+02	3.16E+02	6.81E+02	3.50	2.50	2.50	2.83	Aluminum	15	104ºF	40°C
PEDV	85 A-C	3.16E+03	1.78E+03	3.16E+01	5.62E+02	3.50	3.25	1.50	2.75	Cardboard	60	104ºF	40°C
PEDV	86 A-C	3.16E+02	3.16E+02	1.78E+03	5.62E+02	2.50	2.50	3.25	2.75	Aluminum	60	104ºF	40°C
PEDV	91 A-C	3.16E+01	0.00E+00	3.16E+01	1.00E+01	1.50	0.00	1.50	1.00	Cardboard	360	104ºF	40°C
PEDV	92 A-C	0.00E+00	0.00E+00	0.00E+00	0.00E+00	0.00	0.00	0.00	0.00	Aluminum	360	104ºF	40°C
PEDV	97 A-C	0.00E+00	0.00E+00	0.00E+00	0.00E+00	0.00	0.00	0.00	0.00	Cardboard	1440	104ºF	40°C
PEDV	98 A-C	0.00E+00	0.00E+00	0.00E+00	0.00E+00	0.00	0.00	0.00	0.00	Aluminum	1440	104ºF	40°C
PEDV	103 A-C	0.00E+00	0.00E+00	0.00E+00	0.00E+00	0.00	0.00	0.00	0.00	Cardboard	720	104ºF	40°C
PEDV	104 A-C	0.00E+00	0.00E+00	0.00E+00	0.00E+00	0.00	0.00	0.00	0.00	Aluminum	720	104ºF	40°C
PEDV	109 A-C	0.00E+00	0.00E+00	0.00E+00	0.00E+00	0.00	0.00	0.00	0.00	Cardboard	2160	104ºF	40°C
PEDV	110 A-C	0.00E+00	0.00E+00	0.00E+00	0.00E+00	0.00	0.00	0.00	0.00	Aluminum	2160	104ºF	40°C
PEDV	115 A-C	1.78E+03	5.62E+03	1.78E+03	2.61E+03	3.25	3.75	3.25	3.42	Cardboard	15	122ºF	50°C
PEDV	116 A-C	0.00E+00	0.00E+00	0.00E+00	0.00E+00	0.00	0.00	0.00	0.00	Aluminum	15	122ºF	50°C
PEDV	121 A-C	5.62E+01	3.16E+01	3.16E+01	3.83E+01	1.75	1.50	1.50	1.58	Cardboard	60	122ºF	50°C
PEDV	122 A-C	0.00E+00	0.00E+00	0.00E+00	0.00E+00	0.00	0.00	0.00	0.00	Aluminum	60	122ºF	50°C
PEDV	127 A-C	0.00E+00	0.00E+00	3.16E+01	3.16E+00	0.00	0.00	1.50	0.50	Cardboard	360	122ºF	50°C
PEDV	128 A-C	0.00E+00	0.00E+00	0.00E+00	0.00E+00	0.00	0.00	0.00	0.00	Aluminum	360	122ºF	50°C

PEDV	133 A-C	0.00E+00	0.00E+00	0.00E+00	0.00E+00	0.00	0.00	0.00	0.00	Cardboard	1440	122ºF	50°C
PEDV	134 A-C	0.00E+00	0.00E+00	0.00E+00	0.00E+00	0.00	0.00	0.00	0.00	Aluminum	1440	122ºF	50°C
PEDV	139 A-C	0.00E+00	0.00E+00	0.00E+00	0.00E+00	0.00	0.00	0.00	0.00	Cardboard	720	122ºF	50°C
PEDV	140 A-C	0.00E+00	0.00E+00	0.00E+00	0.00E+00	0.00	0.00	0.00	0.00	Aluminum	720	122ºF	50°C
PEDV	145 A-C	0.00E+00	1.78E+01	0.00E+00	0.00E+00	0.00	0.00	0.00	0.00	Cardboard	2160	122ºF	50°C
PEDV	146 A-C	0.00E+00	0.00E+00	0.00E+00	0.00E+00	0.00	0.00	0.00	0.00	Aluminum	2160	122ºF	50°C

Results of PEDV titrations per surface, contact time and temperature.
